# Investigation of 1200 V SiC MOSFETs’ Surge Reliability

**DOI:** 10.3390/mi10070485

**Published:** 2019-07-18

**Authors:** Huan Li, Jue Wang, Na Ren, Hongyi Xu, Kuang Sheng

**Affiliations:** 1 School of Information & Electrical Engineering, Zhejiang University City College, Hangzhou 310015, China; 2 College of Electrical Engineering, Zhejiang University, Hangzhou 310027, China

**Keywords:** 1200 V SiC MOSFET, body diode, surge reliability, silvaco simulation

## Abstract

In this work, the surge reliability of 1200 V SiC metal-oxide-semiconductor field-effect transistors (MOSFETs) from various manufactures has been investigated in the reverse conduction mode. The surge current tests have been carried out in the channel conduction and non-conduction modes. The experimental results show that the maximum surge currents that the devices can withstand are similar for both cases. It is found that short circuits occurred between the gate and the source in the failed devices. The characteristics of the body diode have also changed after the tests. By measuring the device characteristics after each surge current is applied, it can be concluded that the damages to the gate oxide layer and the body diode occurred only when the maximum surge current is applied. By decapping the failed devices and observing the cross section of the damaged cell, it is found that high temperature caused by excessive current flow through the devices during the surge tests is the main reason for the device failure. Finally, the TCAD simulation of the devices has been carried out to bring insight into the operation of the devices during the surge events.

## 1. Introduction

For decades, silicon material has always been the main semiconductor material used to power electronic devices. However, the performance of silicon devices has approached its theoretical limit determined by the material characteristics. While various applications demand ever-increasing performance from power devices, silicon can no longer meet the requirements of high power, high frequency, high speed and high temperature. Therefore, more attention has been paid to power electronic devices based on wide bandgap semiconductor materials [1,2].

Compared with Silicon, wide bandgap semiconductors SiC, GaN and diamond have the advantages of wide bandgap, high thermal conductivity and high saturation speed. Due to their excellent performance, wide bandgap semiconductors are more suitable for high voltage, high temperature and high switching frequency applications [3,4]. While power devices based on diamond are still in the early research stage [5], some GaN and SiC products are already available in the commercial market. At present, commercial GaN devices are mainly high electron mobility transistors (HEMTs) rated 600 V and below [6,7]. On the other hand, SiC devices are more widely available in voltage ranges between 600 and 3300 V [8,9].

Although wide bandgap semiconductor materials have a lot of advantages, their high cost and poor reliability restrict their large scale commercialization. Current surges are common in the power electronics circuits. For example, the devices have to withstand several folds of the rated current during the startup process of a power factor correction (PFC) circuit or an inverter-fed motor drive. The maximum surge current that a device can withstand is usually referred to as its surge current capability. Excessive surge current will cause irreversible damage to devices, and continuous surge current will also have a negative impact on the device performance. Therefore, surge capability is an important parameter of devices requiring detailed investigation. Among the three wide bandgap semiconductors mentioned above, SiC devices are the most mature. At this point, metal-oxide-semiconductor field-effect transistor (MOSFET) is the SiC commercial product attracts the most attention. Therefore, in this work, surge capability of SiC MOSFETs has been studied.

Until now there have been a number of surge reliability studies of Si diodes [10] and insulated-gate bipolar transistors (IGBTs) [11,12,13]. Considerable research work about the surge reliability of SiC devices has also been carried out in recent years, but the results mainly focused on the reliability of SiC Schottky diodes [14,15,16,17]. On the SiC MOSFET, most reliability work has been carried on under short-circuit conditions. Some researchers have investigated the failure of SiC MOSFETs under short circuit and surge current conditions with a thermal model [18]. Some researchers have tested the maximum surge currents of 1200 V SiC MOSFETs’ body diodes under various conditions [19,20]. The test conditions were varied by changing temperatures, pulse width of surge currents, gate voltages and so on. Some have also compared the surge reliability of SiC MOSFET’s body diodes to those of other Si devices’ body diodes [21,22,23,24]. However, in these works only measurement results were presented. Analysis on the internal causes of the device failure has not been presented.

In this paper, the surge reliability of SiC MOSFETs operating in the reverse conduction mode has been studied. Firstly, several 1.2 kV SiC MOSFETs from several major manufactures has been tested in the channel conduction and non-conduction modes to get the maximum surge currents that the devices could withstand. The performance changes of devices after each surge test were checked to help to understand the process of the failure. In order to find out the failure mechanism, anatomy work has been done to two representative failed devices to identify the damages inside the devices. The results have been analyzed in depth. At last, TCAD simulation has been employed to obtain some insight into the operation of the devices during the surge events.

## 2. Materials and Methods

**Figure 1:**
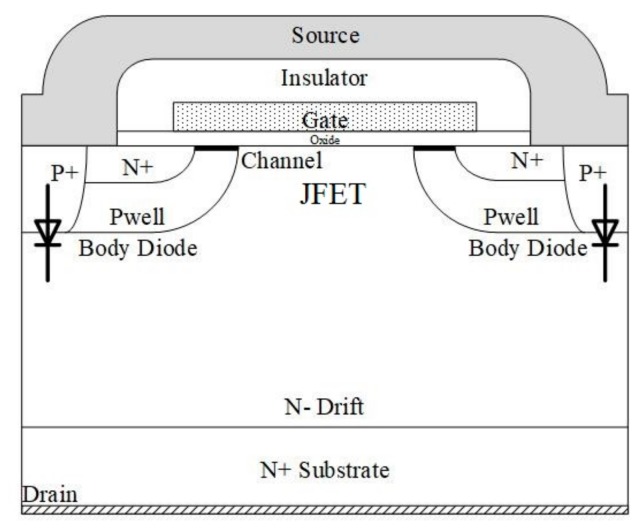
Cell structure of SiC metal-oxide-semiconductor field-effect transistor (MOSFET).

The SiC MOSFETs studied in this paper were vertical N- channel power MOSFETs with a planar gate structure. Shown in Figure 1 is the cell structure of the SiC MOSFET C2M0080120D from Cree Company (Durham, NC, USA). The drain electrode is located at the bottom of the device, in contact with the N+ substrate region. The source electrode and the gate electrode are located on the top of the device, separated by the interlayer insulator. The channel is located in the P− well region, between the N+ source region and the N− drift region. The P+/P− well regions and N− drift region form a PN junction, which is the body diode. The dimension of the regions inside the device has been obtained by SEM. The doping concentrations of the regions have been derived from the device characteristics and the dimension of the regions. The structure parameters of MOSFET are shown in Table 1.

### 2.1. The Reverse Conduction Characteristics of SiC Metal-Oxide-Semiconductor Field-Effect Transistor (MOSFET) 

Until now, there have been a lot of studies on SiC MOSFETs’ body diodes. Their static characteristics are similar to those of the ordinary diodes [25,26,27,28]. 

**Figure 2:**
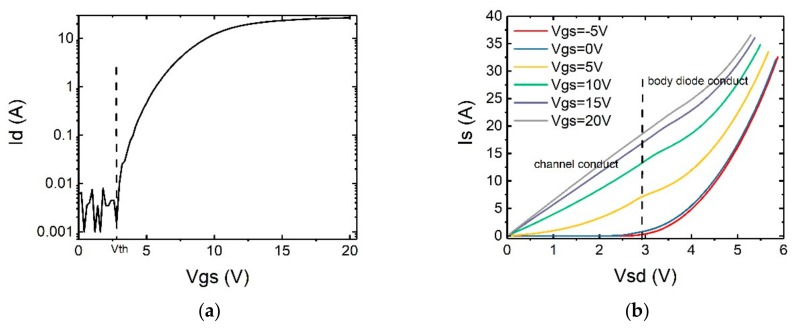
(**a**) Transfer characteristic with V_ds_ = 10 V and (**b**) reverse I-V curves of C2M0160120D from Cree.

In Figure 2a, the transfer characteristics of the SiC MOSFET C2M0160120D manufactured by Cree Company is measured to obtain the threshold voltage and plotted. It can be seen that the MOSFET has a threshold voltage of around 2.6 V. By changing the gate voltages, the reverse I-V curves of the device are tested and the results are shown in Figure 2b. 

In the reverse conduction mode, there are two possible parallel current paths, namely, the body diode and the MOS channel. The body diode only turns on when the voltage across the P+ or P−well/N− drift junction exceeds its turn-on voltage (approx. 2.7 V under room temperature), which explains the I-V curve with V_gs_ = −5 V in Figure 2b. On the other hand, when reverse-biased, the MOS channel opens when V_gd_ exceeds V_th_ and current flows from source to drain. This explains the I-V curves with V_gs_ = 5 V to 20 V where the reverse current of the device increases starting from V_sd_ = 0 V. When V_gs_ = 0 V, the MOS channel starts reverse conducting at V_sd_ = 2.6 V and the body diode starts conducting at V_sd_ = 2.7 V, which gives an I-V curve very similar to that of V_gs_ = −5 V [29]. It is also interesting to note that, for V_gs_ = 5 V to 20 V, the I-V curves start to bend upwards when V_sd_ exceeds 3 V. This is because of the addition of the body current to the existing MOS current. Once turned-on, the current in the body diode increases exponentially with voltage. As a result, the body diode becomes the main conducting path with further increase of the bias voltage.

### 2.2. Setup of the Surge Current Tests

**Figure 3:**
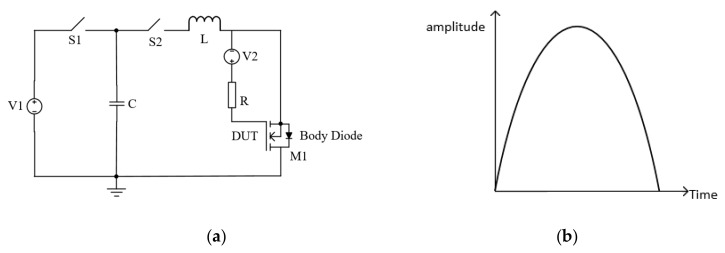
(**a**) Simplified schematic of the surge current test circuit, (**b**) the waveform of the surge current.

The simplified schematic of the surge current reliability test circuit is shown in Figure 3. To test the surge reliability of the device-under-test (DUT), this circuit is operated in the following steps. Firstly, *S*_1_ is closed and *S*_2_ remains open. The voltage source *V*_1_ charges the capacitor *C*. Then, *S*_1_ is open and *S*_2_ is closed. The charge stored in capacitor *C* is discharged through the DUT (*M*1) and the inductor *L*. A large instantaneous current flows through the DUT. The *LC* oscillation circuit is used to generate a sinusoidal surge current. The period of the current was determined by the inductance *L* and capacitance *C*, which could be expressed as:

(1) $$T=2\pi \sqrt{LC}$$

In the tests, the sinusoidal current period is set as 20 ms [30]. The values of *C* and *L* are chosen as: *C* = 2 mF, *L* = 5 mH to satisfy the equation. The magnitude of the sinusoidal current is determined by the following formula:

(2) $$\frac{1}{2}CU^{2}=\frac{1}{2}LI^{2}$$

in which *U* is the voltage of *V*_1_ and *I* is the amplitude of the sinusoidal current flowing through the DUT. During the tests, the amplitude of the surge current is increased gradually by adjusting voltage source *V*_1_.

In this experiment, 1200 V SiC MOSFETs from several major manufacturers were selected for testing. They were C2M0080120D and C2M0160120D from Cree, SCT3160KLGC11 from ROHM (Kyoto, Japan), SCT10N120 from STM (Geneva, Switzerland) and LSIC1MO120E0160 from Lifflefuse (Chicago, IL, USA). The major parameters for the tested devices have been listed in Table 2.

All the devices mentioned above were tested under the reverse conduction state. A half-wave sinusoidal current pulse with a pulse width of 10 ms was used. The amplitude of the current was increased gradually until the device failed. After each test, the device was cooled down to the room temperature before the next test started, so as to prevent the heat generated in the device from affecting the results of the subsequent tests. The surge failure of the device could be determined by observing the distortion of the voltage *V_ds_*.

The maximum surge current capability of each device was obtained from the tests. The tests have been carried out in the channel conduction and non-conduction modes. Therefore, two gate-source voltage biases, 10 V and −3 V, were applied respectively. For every device type and each test condition, three samples were tested.

After the device failed, the static characteristics were measured and compared to the characteristics obtained before the surge currents were applied. These measurements include the transfer characteristic, the reverse I-V characteristics and the resistance between electrodes.

## 3. Results

**Figure 4:**
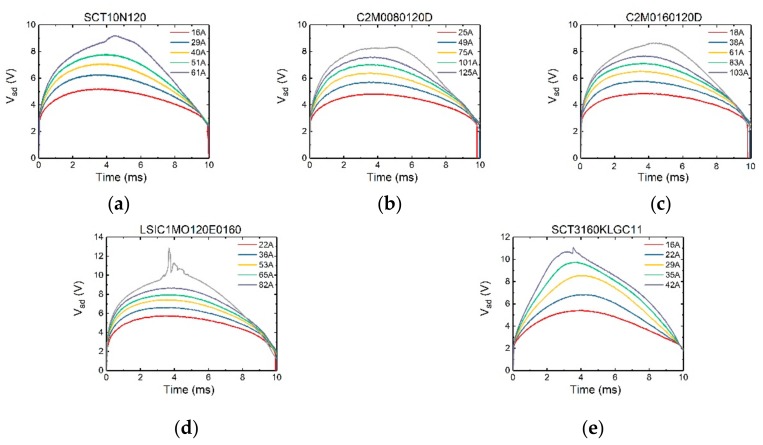
The variation of voltage V_sd_ of (**a**) SCT10N20, (**b**) C2M0080120D, (**c**) C2M0160120D, (**d**) LSIC1MO120E0160, (**e**) SCT3160KLGC11 SiC MOSFETs when varying surge current amplitude with *V_gs_* = −3 V.

**Figure 5:**
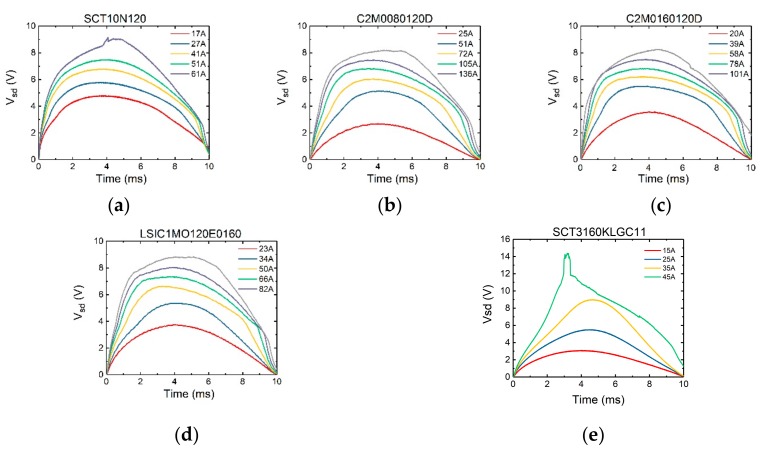
The variation of voltage V_sd_ of (**a**) SCT10N20, (**b**) C2M0080120D, (**c**) C2M0160120D, (**d**) LSIC1MO120E0160, (**e**) SCT3160KLGC11 SiC MOSFETs when varying surge current amplitude with *V_gs_* = 10 V.

The surge test results of devices were shown in Figure 4 and Figure 5. 

In Figure 4, *V_gs_* is set to −3 V. It can be seen that initially there is a sudden rise in the voltage curve. Since with *V_gs_* = −3 V, the current largely flows through the body diode region. The magnitude of the sudden rise corresponds to the on-set voltage of the body diode. Then the voltage increases accordingly with the current. However, it can be seen that the peak of the voltage does not occur when the current reaches its peak value (which is at 5 ms for a 20 ms-period sinusoidal current waveform). It is because in the bipolar mode, the device has a negative temperature coefficient. During each test pulse, as the device is continuously heated up, the voltage peak appears somewhat earlier than the current peak.

In Figure 5, *V_gs_* is set to 10 V. Initially there is no sudden rise of the voltage, since the channel is turned on and it carries all the current. When the voltage *V_sd_* exceeds the on-set voltage of the body diode (~2.7 V), the slope of the voltage curve changes as the body diode starts to conduct. Comparing Figure 4 and Figure 5, the maximum surge currents of the SiC MOSFETs’ in channel conduction and non-conduction modes are very close to each other. This is due to the fact that at *V_sd_* of 6 V and higher (with a likely high junction temperature), the body diode is operating in a bipolar mode with heavy conductivity modulation, making it the dominant current path against the MOS channel. Therefore, whether the channel is conducting or not does not significantly impact the surge current capability of the device. It is also found that the device failure occurs at the time of 3.5 ms in both cases.

The maximum surge currents that the devices can withstand are listed in Table 3. It can be seen that in general the maximum surge current is about 5 times of rated current.

## 4. Discussion

### 4.1. Analysis of Test Result

#### 4.1.1. Comparison of Devices’ Characteristics before and after the Surge Reliability Tests

In this section, the characteristics of the failed MOSFETs have been measured and summarized. According to their behaviors after the tests, the devices can be classified into two groups: The devices in the first group cannot block the reverse voltage anymore, while the devices in the other group still have the reverse voltage blocking capability. Therefore, the following work focuses on two devices representing these two cases respectively: SCT10N120 from STM and C2M0160120D from Cree.

The first failure phenomenon is the short circuit between the gate and the source. During the test, it was found that when the failure occurred, the gate-source voltage dropped to 0 V and a bias voltage could not be applied to the gate. It was preliminary judged that the short circuit occurred between the gate and the source. Therefore, the resistances between the electrodes of MOSFETs before and after the surge failure were tested and shown in Table 4. It can be seen that the resistances before the surge tests are very large and out of the measurement range of the digital multimeter. However, the resistances between the gate and the source of the damaged devices are less than 1 Ω, indicating that the gate is short circuited to the source. In addition, *R_ds_* of the device SCT10N120 after the test is 14 kΩ, which means that damage has also occurred in regions between the drain and the source.

**Figure 6:**
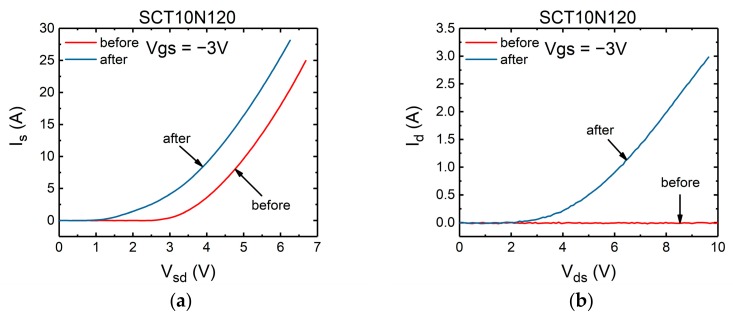
The characteristics of SiC MOSFET’s body diode (SCT10N120) before and after the failure (**a**) forward conduction characteristics, (**b**) reverse blocking characteristics.

**Figure 7:**
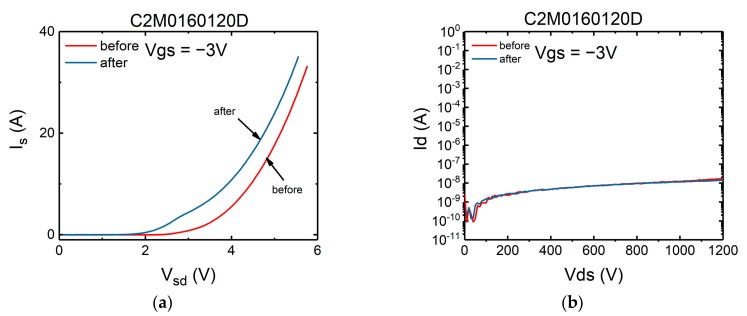
The characteristics of SiC MOSFET’s body diode (C2M1060120D) before and after the failure (**a**) forward conduction characteristics, (**b**) reverse blocking characteristics.

The second abnormal behavior is that the performance of the body diode has changed. In Figure 6 and Figure 7, the forward I-V and reverse blocking characteristics of the failed devices were measured and compared against those before failure.

As shown in Figure 6, for some devices represented by SCT10N120 (STM), the performance of the body diode has changed significantly after the tests, while the devices represented by C2M0160120D (Cree) was not damaged as badly as SCT10N120 (see Figure 7). For both devices, the forward voltage drops of the body diodes have decreased, but the voltages of Cree’s device were reduced less. In terms of the reverse blocking capability, the difference is more significant. Device SCT10N120 has completely lost its reverse blocking capability. However, the Cree device C2M0160120D still exhibits excellent reverse blocking capability.

#### 4.1.2. Static Characteristic Variation of the Tested Device

In the sections above, the results of the surge reliability tests and the behaviors of the failed devices have been presented and summarized. However, device failure did not necessarily occur at the test the maximum surge current was applied. The damage might have accumulated when the surge current was increased gradually. To help to understand the process of the failure and find out the failure mechanisms of the devices, the characteristics of the devices have been measured after every surge current was applied.

The damage of SiC MOSFETs usually occurs in the gate oxide layer, so it is necessary to observe the change of the gate oxide layer’s characteristics. Whether the gate oxide layer is damaged or not can be observed from the change of the transfer curves. Thus, the transfer characteristics have been measured with *V_ds_* = 10 V. The sub-threshold swing is a performance index to measure the switching rate [31]. It is closely related to the interface trap density of gate oxide layer. It represents the variation of the gate voltage required for a ten-fold change in the drain current when the device is operating in the sub-threshold region. It is also called S factor. Smaller S factor corresponds to faster switching rate. It can be calculated from the following equation:

(3) $$S=\frac{dV_{gs}}{dlogI_{d}}$$

The sub-threshold swing is related to the interface trap density *D_it_*, as shown in the following equation:

(4) $$D_{it}=\frac{C_{ox}}{q^{2}}\left(\frac{qs}{\ln\left(10\right)kT}-1\right)-\frac{C_{b}}{q^{2}}$$

**Figure 8:**
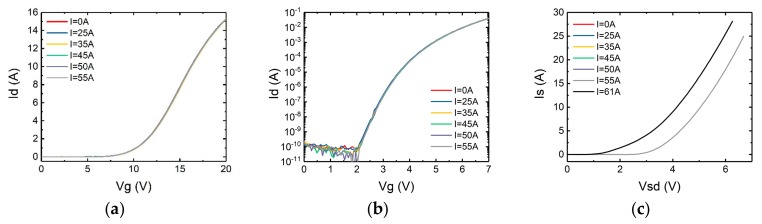
(**a**) Transfer characteristics, (**b**) sub-threshold swing characteristics, (**c**) forward conduction characteristics of the body diode of SCT10N120 after each surge test.

**Figure 9:**
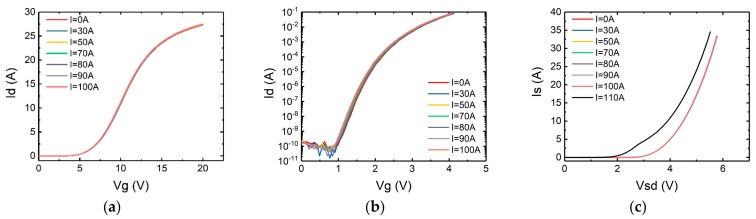
(**a**) Transfer characteristic, (**b**) sub-threshold swing characteristics, (**c**) forward conduction characteristics of the body diode of C2M0160120D after each surge test.

Hence the change of the interface trap density can be derived from the change of sub-threshold swing. In turn the state of gate oxide layer can be deduced by observing the change of the sub-threshold swing characteristics. The results have been measured and shown in Figure 8 and Figure 9. Also included are the forward conduction characteristics of the body diode in which *V_gs_* is set to −3 V to keep the channel off.

It can be seen from the figures that even applying a surge current very close to the maximum surge current the static characteristics of the devices remain nearly unchanged. Hence it can be concluded that the damages to the gate oxide layer and the body diode occurred only at the time the maximum surge current was applied. 

### 4.2. Analysis of Failure Mechanism

During a surge event, the junction temperatures inside the semiconductor devices can reach extremely high values, possibly several times the maximum rated temperature. It was reported that the main failure mechanism for standard silicon diode and transistor chips within module housings, is the melting of the anode-side metallization [32,33,34].

**Figure 10:**
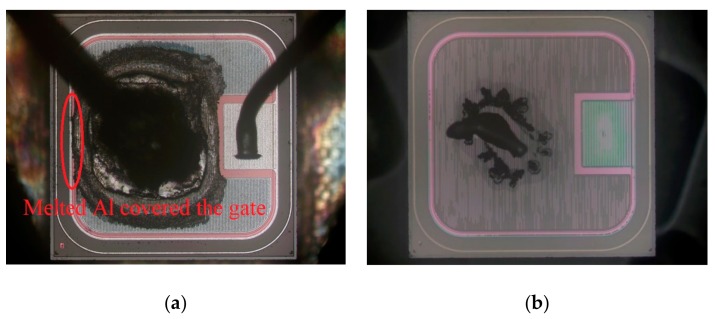
The anatomy results of STM device SCT10N120 (**a**) decapping, (**b**) removing layers to the substrate layer.

**Figure 11:**
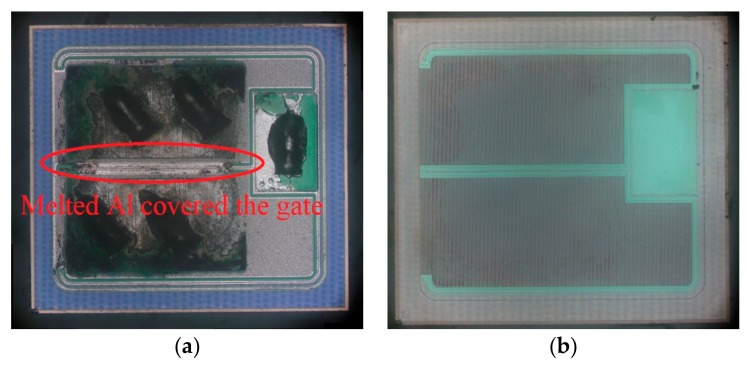
The anatomy results of Cree device C2M0160120D (**a**) decapping, (**b**) removing layers to the substrate layer.

To investigate the failure mechanisms of these tested SiC MOSFET devices, anatomy work has been carried out. The following two devices were examined, namely, SCT10N120 (STM) and C2M0160120D (Cree). The anatomical results of the damaged devices are shown in Figure 10 and Figure 11. 

Figure 10a is the image of the decapped STM device SCT10N120. The burn mark of the device is very obvious. Removing the layers to the substrate, there is still apparent damage in the substrate layer (Figure 10b). The burned area is concentrated near the bonding wires, indicating that the burnout was caused by the excessive current flowing through the bonding wires.

Figure 11a is the image of the decapped Cree device C2M0160120D. There are also severe burn marks. Compared with the results of SCT10N120, the burn areas of Cree device are more uniform and the degree of burn is lighter. It was found that the Cree device has more bonding wires (four wires) than the STM device (one wire). Therefore, it is possible that multiple bonding wires can spread out current, thus lower the maximum junction temperature and improve the surge performance of devices.

In addition, it is found that the melted Al covers the gate, as marked in the figures. This indicates that Al might have penetrated into the gate and causing the short circuit between the gate and the source. 

**Figure 12:**
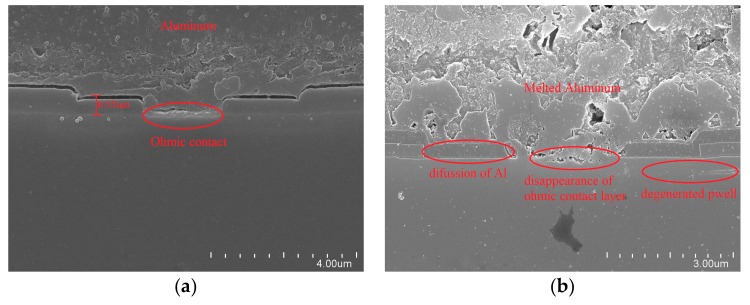
SEM images of SCT10N120 (**a**) before, (**b**) after the device failure.

**Figure 13:**
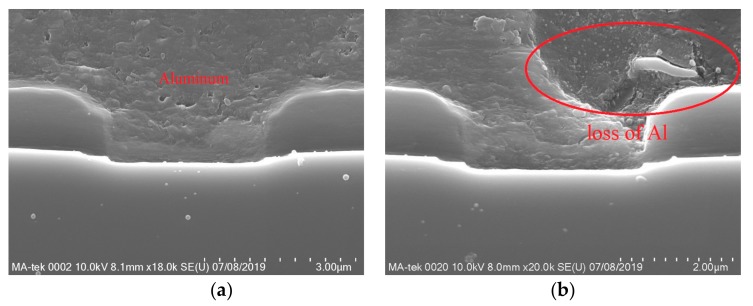
SEM images of C2M0160120D (**a**) before, (**b**) after the device failure.

From the anatomical results, the high temperature caused by excessive current is the main reason for the device failure. The high temperature caused burnout in many areas inside the device and eventually led to the device failure. In order to understand the process of the failure more clearly, the cross section of the damaged cell was cut and observed by SEM. The results are shown in Figure 12 and Figure 13.

Device SCT10N120 has been damaged more severely than device C2M0160120D. By comparing the SEM images of SCT10N120 taken before and after the failure, a few different types of device damages are possible.

#### 4.2.1. Aluminum Melted and Diffused into the Interlayer Dielectric

Aluminm (Al) is used to form electrodes in these devices. Firstly, it is observed that Al was melted inside the failed devices. Secondly, it is found that the interlayer dielectric became thinner and its depth is changed from 0.55 µm to 0.36 µm, which indicates that Al may has eroded the interlayer dielectric. 

A high temperature can melt Al which could diffuse into the insulator and cause the short circuit of the gate and source. The process is described by the following equation:

(5) $$2\mathrm{Al}+\frac{3}{2}{\mathrm{SiO}}_{2}\to {\mathrm{Al}}_{2}O_{3}+\frac{3}{2}\mathrm{Si}$$

As mentioned in some literature, the product of the above reaction, Si, rapidly dissolves or migrates into Al, leaving fine voids, (−)_Al2O3_, in Al_2_O_3_.

(6) $$\mathrm{Al}+\mathrm{Si}\to \mathrm{Al}:\mathrm{Si}+{\left(-\right)}_{{\mathrm{Al}}_{2}O_{3}}$$

Then, Al will self-diffuse into the voids and fills them,

(7) $$\mathrm{Al}+{\left(-\right)}_{{\mathrm{Al}}_{2}O_{3}}\to {\left(\mathrm{Al}\right)}_{{\mathrm{Al}}_{2}O_{3}}$$

where (Al)_Al2O3_ represents the Al that occupies the Al_2_O_3_ voids. Thus, the Al can react again with fresh SiO_2_ (silicate glass) at the bottom of the Al_2_O_3_ voids in the process described by Equation (6) [35,36,37].

In addition, white spots appeared in the interlayer dielectric, indicating that Al has diffused into the interlayer dielectric. It also caused the short circuit of the gate and source.

#### 4.2.2. Ohmic Contact Layer Disappeared

As shown in Figure 12a, there is an ohmic contact layer between the source metal and N+ source area. In Figure 12b, this ohmic contact layer disappears and the boundary between the source metal and N+ source area disappears.

For NMOS, Ni is generally used as an ohmic contact material [38,39]. The ohmic contact layer is formed by rapid annealing at high temperature after the deposition. Because Ni can easily react with SiC to form silicide at high temperature, NiSi and Ni_2_Si are the main components in the ohmic contact layer.

Previously Hökelek and Robinson reported that Al started to react with NiSi at 400 °C by decomposing it and forming NiAl_3_ and Si [40], as shown in the following equation:

(8) $$3\mathrm{Al}+\mathrm{NiSi}\;\to {\;\mathrm{NiAl}}_{3}+\mathrm{Si}$$

In reference to the findings of Bartur and Nicolet [41], the disappearance of Ni_2_Si can be described as the result of an alternate process consisting of the thermodynamically feasible reaction:

(9) $$6\mathrm{Al}+{\mathrm{Ni}}_{2}\mathrm{Si}\to 2{\mathrm{NiAl}}_{3}+\mathrm{Si}$$

The eutectic reaction of Si and Al (Equation (9)) enables Al to react with the ohmic contact layer continuously, eventually lead to the disappearance of the ohmic contact layer.

(10) Al+Si→Al:Si

#### 4.2.3. Al Penetrated into SiC

The ohmic contact layer can prevent contact between Al and SiC. However, with the disappearance of the ohmic layer, Al penetrated into SiC regions. In Figure 12b, there is a discontinuous white line at 0.42 µm below the gate showing that Al has penetrated into the device. It is located at the boundary between the Pwell region and the N- drift region. It would affect the contact barrier of the body diode. Thus, the blocking performance of body diode would be affected.

In comparison, device C2M0160120D only exhibits the loss of Al, indicating Al has melted during the test. However, no penetration of Al into SiC regions has been found. That explains why the body diode of C2M0160120D can still have the reverse blocking capability after the test.

Therefore, the above analysis further reveals the failure regions and mechanisms of the device. When a large surge current flowed into the device, the device quickly heated up. The metal of the source electrode melted, eroded the interlayer dielectric then made the gate and the source contact each other. This eventually led to the short circuit between the gate and the source. Furthermore, the melted Al penetrated into the ohmic contact layer and made the ohmic contact layer disappear. Penetration of the Al into the body diode P/N junction also compromises its blocking capability.

### 4.3. Analysis of Simulation Result

In order to verify the findings of the experiments, finite element numeric simulation of the device has been carried out.

#### 4.3.1. Simulation Setting

The simulation was completed by Silvaco TCAD, the device structure was generated by Devedit, and the surge simulation was completed by Mixmode. The structure of the SiC MOSFET C2M0080120D is shown in Figure 1. The structure parameters of the device are listed in the Table 1. The physical models used in simulation are shown in Table 5.

**Figure 14:**
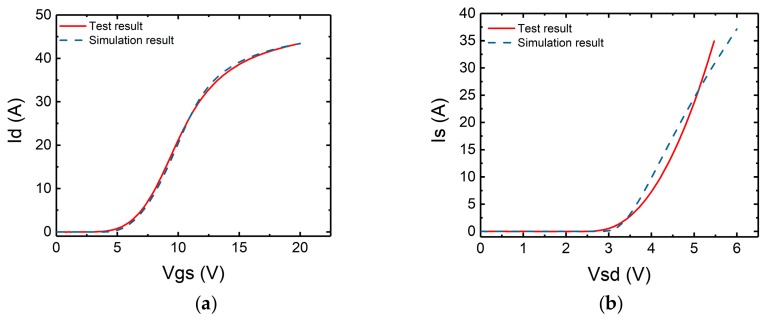
Comparison of (**a**) transfer characteristics, (**b**) body diode characteristics, between simulation and test result.

To verify the simulation setting, the transfer characteristic of the device has been simulated and compared with the experimental results in Figure 14a. It can be seen that the curves match each other. Also shown in Figure 14b is the simulation and experimental results of the body diode forward conduction characteristics. The results are very similar.

#### 4.3.2. Surge Simulation Results and Analysis

Based on the device structure parameters listed in Table 1, the device was simulated and results were obtained. The input current was a sinusoidal current, with a 20 ms period.

##### The Channel Non-Conducting Mode

Atlas is not capable of directly simulate the device failure discussed in the previous sections, as it is mainly caused by some chemical reactions and the melting movement of aluminum at high temperature. Nevertheless, it is possible to simulate the temperature distribution inside the device when the surge occurs. 

**Figure 15:**
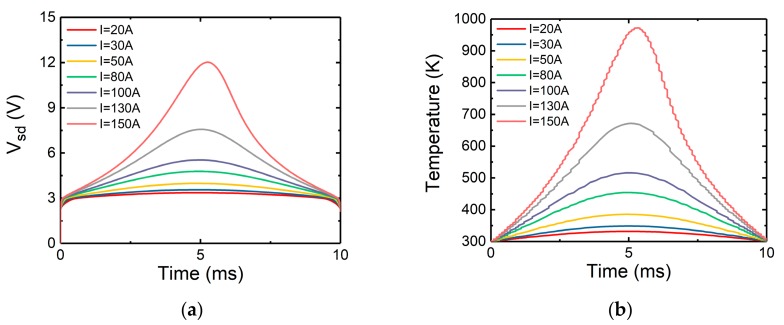
When the channel is non-conducting, the simulated (**a**) source-drain voltage, (**b**) maximum temperature, with time.

Mixed mode thermal simulation of the device has been carried out, and the results were obtained and shown in Figure 15.

Plotted in Figure 15a is the variation of *V_sd_* with time inside the device. Initially, there is a voltage jump. This phenomenon matches the experimental results, as shown in Figure 4. In the channel off mode, only the body diode is conducting, and the body diode will not conduct until the threshold voltage is reached. Therefore, there is a voltage jump in the beginning. 

Figure 15b shows the change of maximum temperature inside the device with time under different surge current conditions. It can be seen that the trend of the maximum temperature is roughly consistent with that of the voltage. When the current peak value increases, the maximum temperature rises gradually. It can also be found that the temperature rises faster with the increase of current peak value.

##### The Channel Conduction Mode

**Figure 16:**
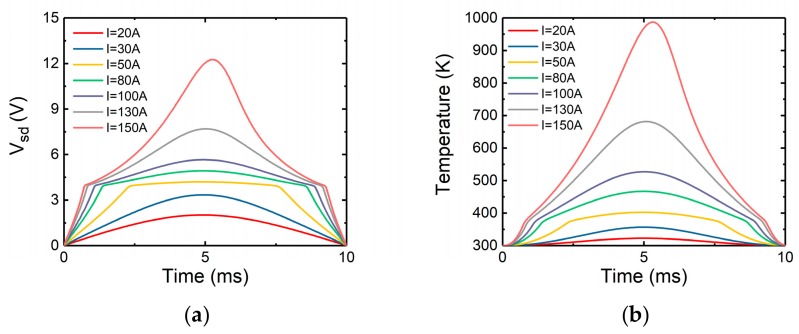
When the channel is conducting, the simulated variation of (**a**) source-drain voltage, (**b**) maximum temperature versus time.

The simulated curve of *V_sd_* with time in the channel conduction mode is shown in Figure 16a. In Figure 16a, there is an obvious turning point on the voltage curve. In the channel conduction mode, at low current peak value the current flows through the channel. However, as the surge current increases, *V_sd_* exceeds the body diode threshold voltage and the body diode conducts current as well. In the experiments, this turning point is not so obvious due to the influence of the parasitic capacitance and inductance. Shown in Figure 16b is the variation of internal maximum temperature with time in the channel conduction mode. The curve is similar to the case when the channel is not conducting.

**Figure 17:**
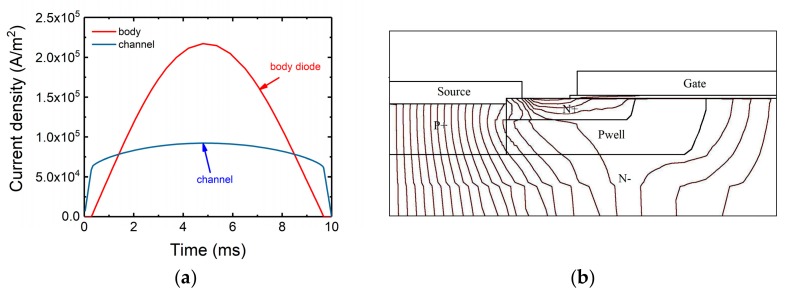
(**a**) Current density of body diode region and channel versus time. (**b**) Current flowlines in device when time = 5 ms.

The current densities in the body diode region and the channel when the channel was conducting have been simulated to find out the reasons why channel conduction had little effect on the device surge tolerance. The simulation results are shown in Figure 17. At low current peak value, the current flows through the channel only. When the on-set voltage of body diode is reached, a portion of the current was transferred to the body diode region. It can be noticed that the slope of the current density flowing through the channel is decreased. Finally, the current density in the body diode becomes greater than the current density in the channel. Shown in Figure 17b are the current flowlines inside the device at t = 5 ms. The number of current flowlines is proportional to the current density. It can be seen that the number of the current flowlines in the body diode region is much more than that in the channel. From Figure 17, it is found that when the current is large enough, the current mainly passes through the body diode region.

**Figure 18:**
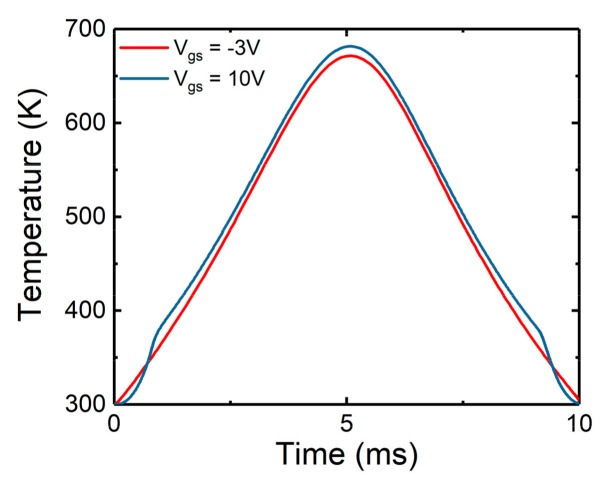
Comparison of the maximum temperature under channel conduction and non-conduction modes when I_s_ = 130 A.

It is found in the previous sections that the surge failure in this study is mainly caused by the high junction temperature. The maximum junction temperatures versus the time under channel conduction and non-conduction modes are compared in Figure 18. It can be seen that the maximum temperatures on conduction mode are slightly higher than that on the non-conduction mode. Therefore, it can be concluded that no matter the channel is conducting or not, the maximum junction temperatures inside the device are similar. As a result, the surge tolerance is approximately equal. This is mainly due to the fact that, when the current is big enough, the body diode current dominates against the MOS channel current. It also can be seen that, with the increase of the current, the maximum temperature inside the device could exceed the melting point of Al (933 K) and lead to the melting of Al.

## 5. Conclusions

In summary, the reverse conduction surge reliability of several 1200 V SiC MOSFETs has been tested in the channel conduction and non-conduction modes in this paper. Device failures under these circumstances have been investigated in detail. Two types of failure characterized by different behavior after the failure have been found. It is demonstrated that during the surge event the excessive heat causes the melting of Aluminum and its subsequent reaction with and penetration into the SiC device and dielectric layers. Open-cover anatomy and 2-D numerical simulation confirms such mechanisms.

By increasing the surge current gradually until failure, it is found that the maximum surge currents that the devices can withstand are similar for both channel conduction and non-conduction modes.

The performance change of the device after failure is mainly reflected in two aspects: The first is the short circuit between the gate and the source, the other is the performance change of the body diode. Although the threshold voltages of the body diodes in all devices have been lowered after the failure, the reverse blocking characteristics of the body diodes are different. The reverse blocking capability of Cree’s devices represented by C2M0160120D remain essentially unchanged, while other devices represented by SCT10N120 lost their reverse blocking capability after the surge failure. Then, the static characteristics of the device have been tested after each surge current was applied to help understanding the process of the failure. It is found that almost no damage occurred to the gate oxide and the body diode region until the maximum surge current was applied.

The open-cover anatomy of the two devices has been carried out. Both devices have burn marks, but the degree of damage is lighter for the Cree device. Increasing the number of bonding wires can spread the current more evenly across the chip and relieve damage caused by high temperature. By observing the cross section of the damaged cell, it is found that when the surge failure occurs, the source metal melts and penetrates into the interlayer dielectric and the P-well region, leading to the failure of the device.

Silvaco finite element numerical simulation has been employed to study the variation of the internal temperature and current density during the surge tests. In the simulation, it is found that when the surge current reaches a large value, the current mainly flows through the body diode. Therefore, whether the channel conducts or not has limited effect on the surge capability. This conclusion is consistent with the experimental results.

## Figures and Tables

**Table 1:** Structure parameters of the metal-oxide-semiconductor field-effect transistor (MOSFET) shown in Figure 1.

Region	Numerical Value	Unit
Doping concentration in P−well region	Surface $7\times 10^{16}$, internal peak 1 $\times 10^{18}$	cm^−3^
Depth of P−well region	0.5	μm
Doping concentration in N− drift region	$8.85\times 10^{15}$	cm^−3^
Depth of N- drift region	10	μm
Doping concentration in N++ substrate region	$1\times 10^{19}$	cm^−3^
Depth of N++ substrate region	170	μm
Doping concentration in N+ region	$3\times 10^{18}$	cm^−3^
Depth of N+ region	0.2	μm
Doping concentration in P+ region	$1\times 10^{19}$	cm^−3^
Length of channel	0.96	μm
Thickness of gate oxide layer	0.05	μm
Length of junction field-effect transistor (JFET) region	2.4	μm

**Table 2:** Device parameters from the datasheets.

	*V_ds_* (V)	*R_ds(on)_* (mΩ)	*I_ds_* (A)/ Body Diode *I_F_* (A)	*V_gs_* (V)	*V_th_* (V)
SCT10N120	1200	500	12/12	−10/25	5.5
C2M0080120D	1200	80	36/36	−5/20	2.8
C2M0160120D	1200	160	19/19	−5/20	3
LSIC1MO120E0160	1200	160	22/22	−5/20	3.6
SCT3160KLGC11	1200	160	17/17	−4/22	5

**Table 3:** Maximum surge current that a device can withstand.

	*I_ds_* (A)/ Body Diode *I_F_* (A)	*R_ds(on)_* (mΩ)	Maximum Surge Current (A)	Maximum Surge Current/Rated Current
SCT10N120	12/12	500	61	5
C2M0080120D	36/36	80	155	4.3
C2M0160120D	19/19	160	105	5.5
LSIC1MO120E0160	22/22	160	95	4.3
SCT3160KLGC11	17/17	160	42	2.5

**Table 4:** Measured resistances between electrodes before and after surge tests.

Device	*R_gs_* (Ω)	*R_ds_* (Ω)
Before the tests	>100 M	>100 M
SCT10N120, after tests	0.4	14 k
C2M0160120D, after tests	0.08	>100 M

**Table 5:** Physical models used in simulation.

Model	Model Used in Simulation	Notes
Carrier Statistics Models	Fermi-Dirac	Reduced carrier concentrations in heavily doped regions (statistical approach).
	Bandgap Narrowing(BGN)	Important in heavily doped regions. Critical for bipolar gain. Use Klaassen Model.
Mobility Models	Lombardi (CVT) Model	Complete model including N, T, E//, and E_┴_ effects. *
Recombination Models	Shockley-Read-Hall(SRH)	Uses fixed minority carrier lifetimes.
	Auger	Direct transition of three carriers. Important at high current densities.
Impact Ionization Model	Selberherr’s Model (Impact selb)	Recommended for most cases. Includes temperature dependent parameters.

* T is lattice temperature, N is the dopant concentration, E// is parallel electric field, and E_┴_ is perpendicular electric field.
